# Microscopic tumor spread beyond (echo)endoscopically determined tumor borders in esophageal cancer

**DOI:** 10.1186/s13014-019-1419-5

**Published:** 2019-12-04

**Authors:** Melanie Machiels, Maurits L. van Montfoort, Nikki B. Thuijs, Mark I. van Berge Henegouwen, Tanja Alderliesten, Sybren L. Meijer, Jeanin E. van Hooft, Maarten C. C. M. Hulshof

**Affiliations:** 1 Department of Radiation Oncology, Amsterdam UMC, University of Amsterdam, Meibergdreef 9, Amsterdam, Netherlands; 2 Department of Pathology, Amsterdam UMC, University of Amsterdam, Meibergdreef 9, Amsterdam, Netherlands; 3 Department of Surgery, Amsterdam UMC, University of Amsterdam, Meibergdreef 9, Amsterdam, Netherlands; 4 Department of Gastroenterology and Hepatology, Amsterdam UMC, University of Amsterdam, Meibergdreef 9, Amsterdam, Netherlands

**Keywords:** Esophageal cancer, Microscopic tumor spread, Clinical target volume, Endoscopy, Fiducial markers, Radiotherapy

## Abstract

**Objective:**

The microscopic tumor spread (MS) beyond the macroscopic tumor borders of esophageal tumors is crucial for determining the clinical target volume (CTV) in radiotherapy. The question arises whether current voluminous CTV margins of 3–5 cm around the macroscopic gross tumor volume (GTV) to account for MS are still accurate when fiducial markers are used for GTV determination. We aimed to pathologically validate the use of fiducial markers placed on the (echo)endoscopically determined tumor border (EDTB) as a surrogate for macroscopic tumor borders and to analyse the MS beyond EDTBs.

**Methods:**

Thirty-three consecutive esophageal cancer patients treated with neo-adjuvant chemoradiotherapy after (echo)endoscopic fiducial marker implantation at cranial and caudal EDTB were included in this study. Fiducial marker positions were detected in the surgical specimens under CT guidance and demarcated with beads, and subsequently analysed for macroscopic tumor spread and MS beyond the demarcations. A logistic regression analysis was performed to determine predicting factors for MS beyond EDTB.

**Results:**

A total of 60 EDTBs were examined in 32 patients. In 50% of patients no or only partial regression of tumor in response to therapy (≥Mandard 3) or higher was seen (i.e., residual tumor group) and included for MS analysis. None had macroscopic tumor spread beyond EDTBs. In the residual tumor group, only 20 and 21% of the cranial and caudal EDTBs were crossed with a maximum of 9 mm and 16 mm MS, respectively. This MS was corrected for each individual determined contraction rate (mean: 93%). Presence of MS beyond EDTB was significantly associated with initial tumor length (*p* = 0.028).

**Conclusion:**

Our results validate the use of fiducial markers on EDTB as a surrogate for macroscopic tumor and indicate that CTV margins around the GTV to compensate for MS along the esophageal wall can be limited to 1–1.5 cm, when the GTV is determined with fiducial markers.

## Introduction

Radiotherapy (RT) with concurrent chemotherapy plays an important role in the treatment of patients with operable or inoperable esophageal cancer [1, 2]. Modern radiation techniques can deliver radiation dose with high precision to the target volume, consequently precise target volume localization is of utmost clinical importance.

In the past years, improvements in gross tumor volume (GTV) determination were introduced focusing on exact demarcation of the GTV. 18-F-Fluorodeoxyglucose positron emission tomography/computer tomography (FDG-PET/CT) scans are nowadays often used to aid in GTV delineation [3]. However, the GTV can be more easily determined with the aid of fiducial markers placed on the (echo)endoscopically determined tumor borders (EDTB), resulting in a significant delineation variation reduction [4]. In the absence of fiducial markers, a very large delineation variation in longitudinal direction was seen, resulting in a bulky CTV-to-PTV margin [4]. Furthermore, with other research focusing on measurement-driven planning target volume (PTV) definitions, the PTV can be tailored to individual patients’ set up uncertainties [5, 6].

It is known that beyond the macroscopic tumor borders that are visible on clinical imaging, esophageal cancer can exhibit microscopic tumor spread (MS). Thus, for adequate radiation coverage the GTV is expanded by an empirically defined margin to the so-called clinical target volume (CTV), encompassing both macro- and microscopic tumor. The CTV is still defined using non-individualized population-based empirical findings and is more often based on the clinician’s experience and institutional convention instead of on a patient-specific basis. Currently, a voluminous margin of 3–5 cm in craniocaudal (CC) direction around the GTV is recommended based on a few, mostly outdated studies [7–9]. These studies are not based on modern pathological analysis techniques, nor used current state-of-the-art diagnostic techniques to facilitate GTV determination such as fiducial markers placement on the EDTB. The question arises whether the current voluminous margins around the GTV to account for MS are still accurate when the GTV is determined by means of (echo)endoscopy. This lack of knowledge may lead to unnecessary radiation of healthy tissue, or to inadequate coverage of tumor extension.

In the present study, we aimed to validate the use of fiducial markers placed on the EDTBs as a surrogate for macroscopic tumor borders with histopathological confirmation and to investigate MS beyond the EDTBs in esophageal cancer patients that underwent neo-adjuvant chemoradiotherapy (nCRT).

## Materials and methods

### Patients

Thirty-three consecutive esophageal cancer patients treated with nCRT undergoing an esophagectomy with gastric tube reconstruction were prospectively included in this study. Inclusion criteria were; (1) completed neo-adjuvant treatment, (2) absence of metastatic disease on pre-operative FDG-PET/CT, (3) fit for surgery, (4) presence of at least one fiducial marker on the EDTB on pre-operative imaging. The study was approved by the institutional review board and was registered under the number NL7683.

### Marker placement on EDTB

All patients underwent before start of treatment, an additional endoscopy and/or linear-array echo-endoscopy (EUS) under midazolam or propofol-based sedation. Procedures were performed by experienced endosonographers. Each patient had at least two flexible coil-shaped gold markers (5-10 mm long with diameter of 0.35 mm; Visicoil, Core Oncology, Santa Barbara, California) implanted (i.e., one exact at the cranial respectively caudal tumor border), as is clinical practice at our center. A third fiducial in the middle of the tumor was optional. The procedure was reported in detail in an earlier study [10]. The goal was to place the fiducial markers exactly on the cranial and caudal EDTB, although this was not always feasible (e.g., due to stenosis). Because the fiducial marker position was aimed to indicate true gross tumor extent, the distance between the placed fiducial marker and the EDTB was measured under fluoroscopy; the EDTB was indicated by the tip of the (echo)endoscope after marker implantation with fluoroscopy. This distance between the fiducial marker and the EDTB was documented.

### Treatment

All patients were treated with intensity-modulated RT (IMRT) or volumetric modulated arc therapy (VMAT) depending on time of inclusion. The radiation dose was 41.4 Gy in 23 fractions [11]. The GTV was delineated on the planning CT scan using all available resources, including data of diagnostic (FDG-PET/)CT scans, (echo)endoscopic reports, and fiducial markers on EDTBs. If pathological lymph nodes were present, these were delineated as a separate GTV, referred to as GTV_nodal_. The CTV was generated by extending the GTV in the CC direction with a 35 mm margin. When the tumor extended into the cardia, a 20 mm margin in the caudal direction sufficed in the cardiac region. Peripherally the CTV needed to cover the peri-esophageal fatty tissue and regional lymph node stations. In case of pathological lymph nodes, an isotropic CTV margin of 5 mm was taken around the GTV_nodal_ (i.e., CTV_nodal_). If this CTV_nodal_ was located beyond the CTV, the CTV was extended, encompassing all intermediate lymph node stations and peri-esophageal fatty tissue, in order to completely cover the CTV_nodal_. The CTV to planning target volume (PTV) margin was 10 mm in all directions. Daily cone beam CT (CBCT) scans were acquired and used for bony anatomy-based patient setup correction.

RT was combined with weekly paclitaxel (50 mg/m2) and carboplatin (AUC2 mg/ml/min) [3, 6]. After an 8–14 weeks interval, a pre-operative FDG-PET/CT scan was performed. If no metastatic disease was present, a transthoracic or transhiatal esophagectomy with lymphadenectomy was performed depending on patient characteristics. All operations were performed by two surgeons.

### Histopathology

**Fig. 1:**
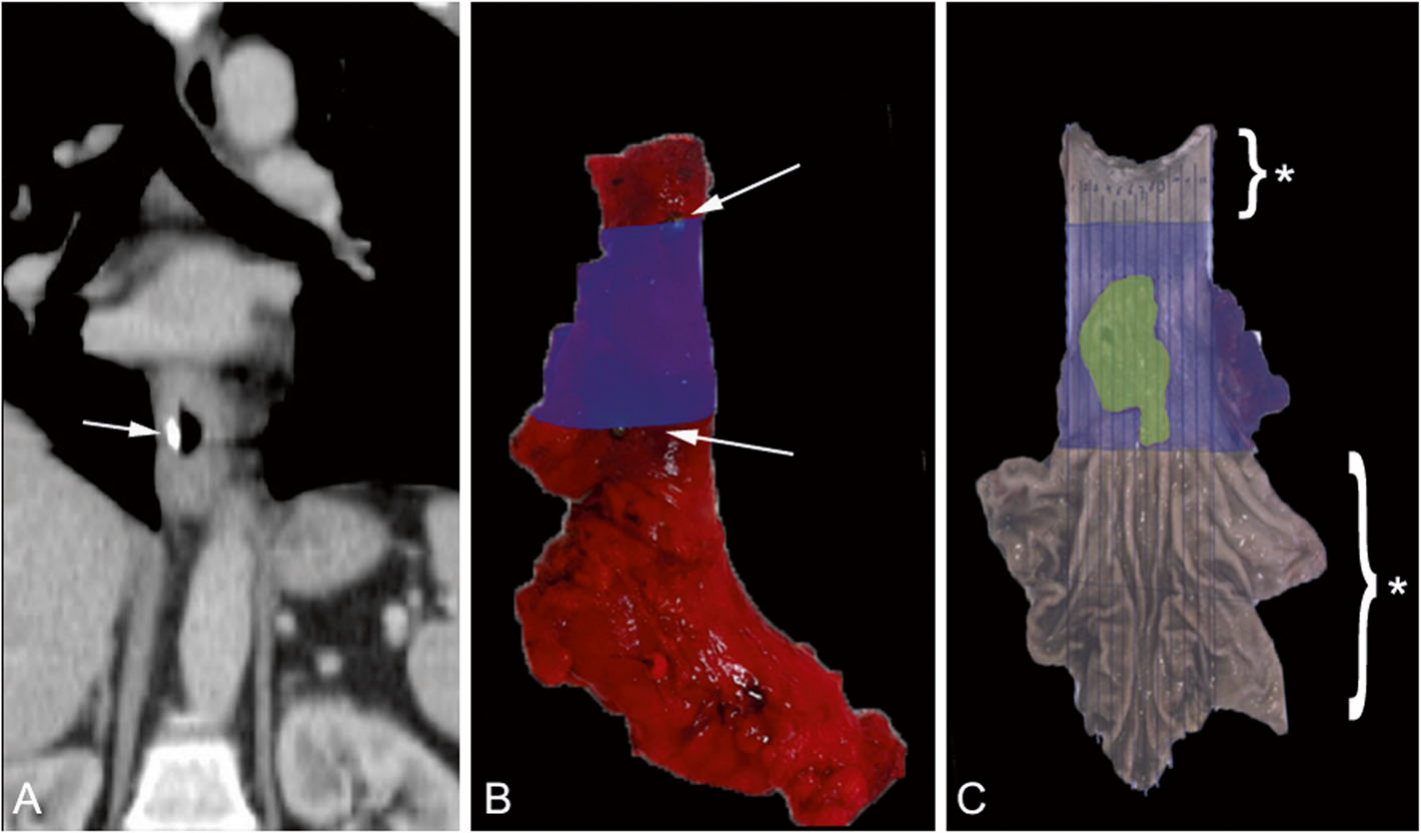
**a**: Pre-operative CT scan of patient 3 in coronal view with caudal fiducial marker visible (indicated by the arrow). **b**: Fresh, unfixed esophageal specimen after surgical removal of patient 3. Blue inked area: (echo)endoscopically determined tumor area between both (echo)endoscopically determined tumor borders. Arrows indicate fiducial marker positions cranially and caudally, demarcated externally with green beads. **c**: Interior of the esophageal specimen of patient 3, after longitudinal opening and fixation. Blue inked area: (echo)endoscopically determined tumor area. Green overlay: area with residual tumor (≥Mandard 3). Asterisk indicates area examined for macroscopic and microscopic tumor spread

The surgical specimens of all patients were collected from the operation room, and fiducial marker positions were detected under CT guidance. If fiducial markers were placed exactly on the EDTB, their location was directly demarcated with beads on the exterior of the specimen (Fig. 1). If fiducial markers were placed at a certain distance from the EDTB, first the contraction rate was determined to deduct the ex-vivo distance of the fiducial from the EDTB. This calculated EDTB was demarcated with beads.

The contraction rate of the esophagus takes the natural contraction of the specimen after excision into account. In our study, the contraction rate was defined as the distance projected on the longitudinal axis - in the center of the esophagus - between the outer fiducial markers (i.e., cranial and caudal) on post-operative CT imaging divided by the distance measured on the last available pre-operative imaging between the outer fiducial markers, when no cardiac involvement was present. In case of cardiac involvement, the third fiducial marker at the middle of the tumor was also incorporated in the contraction rate calculation, overcoming effect of the esophageal curvature towards the cardia.

When an outer fiducial marker was lost, the remaining two fiducials were used to calculate the individual contraction rate. When only one fiducial marker was present, no contraction rate calculation could be performed.

After demarcation of the EDTB under CT guidance on the exterior of the specimen, it was brought to the pathology department for further analyses. The specimen was pinned to a paraffin board before processing, to prevent *processing* contraction, as is standard protocol. The absence of processing contraction was verified in a subset (*n* = 10) of patients, by measuring the distance between the demarcated EDTB again after formalin fixation.

The area between the EDTBs of the individual surgical specimens was stained with a color (Fig. 1b). Subsequently, all resection specimens were evaluated using a standard protocol described earlier [12]. If no macroscopically identifiable tumor was present, lesions such as an ulcer, scar, or an irregular area covered by mucosa were completely embedded, together with surrounding tissue in order to be able to adequately judge the presence of residual tumor and therapy effects. Tumor response to nCRT was evaluated using the 5-tiered Mandard classification, which is based on the ratio of microscopically viable residual tumor cells in relation to the area of fibrosis [13]. Only specimens with Mandard 3–5 were used for MS analysis.

Beyond the cranial and caudal EDTB the esophagus was embedded transversally over the complete width, in order to inspect thoroughly the presence of MS. In the complete/partial responding group, extent of regressional changes (e.g., mucinous lakes, keratin pearls, and/or foreign body giant cell reactions) were also described with respect to the EDTB.

### Follow-up

After resection, routine follow-up was performed every 3 months in the first year and every 6 months in the second year, followed by annual evaluations. Radiological examinations based on clinical suspicion of recurrent disease was standard care for all patients.

### Statistical analysis

Patient characteristics were summarized using descriptive statistics. To identify prognostic factors for MS, univariate logistic regression analyses were performed. Multivariate analyses were performed, entering the parameters of influence on outcome according to the univariate analysis (defined as those with *p*-value < 0.1), using backwards selection. A *p*-value of < 0.05 was considered significant.

Disease-free survival (DFS) and overall survival (OS) time were calculated from the first day of nCRT according to the Kaplan-Meier method.

Statistical analyses were performed using the Statistical Package for Social Sciences, version 24.0 software (SPSS, Chicago, IL).

## Results

Of the 33 patients, 16% had squamous cell carcinoma (SCC) and 84% had adenocarcinoma (AD). Twenty-seven patients had fiducial markers placed exactly on the EDTB, as determined under fluoroscopy. Six patients had at least 1 fiducial marker placed at a certain distance from the EDTB. Patient- and tumor characteristics are listed in Table 1.

**Table 1:** Patient, tumor, and fiducial marker implantation characteristics

	*N* = 32 (%)	Microscopic tumor beyond EDTB *N* = 5 (%)
Patient characteristics		
Gender		
Female	11 (34)	1 (20)
Male	21 (66)	4 (80)
Age (years)		
< 60	5 (16)	1 (20)
> 60	27 (84)	4 (80)
Tumor characteristics		
Clinical T-classification		
cT1	2 (6)	1 (20)
cT2	15 (47)	3 (60)
cT3	15 (47)	1 (20)
cT4	0	0 (0)
Clinical N-classification		
cN0	18 (56)	3 (60)
cN1	10 (31)	2 (40)
cN3	4 (13)	0 (0)
Histology		
Adenocarcinoma	24 (75)	4 (80)
Squamous cell carcinoma	8 (25)	1 (20)
Location		
Mid-thoracic	6 (19)	1 (20)
Distal	19 (59)	3 (60)
GEJ	7 (22)	1 (20)
Cardiac involvement		
Yes	13 (41)	2 (40)
No	19 (59)	3 (60)
Implantation characteristics		
Technique		
EUS	10 (32)	1 (20)
Endoscopy	19 (59)	3 (60)
Combination	3 (9)	1 (20)
Fiducial on EDTB?		
Yes	26 (81)	4 (80)
No	6 (19)	1 (20)

Patient without fiducial markers on pre-operative imaging excluded from table

*Abbreviations*: *EDTB* (echo)endoscopically determined tumor border, *GEJ* Gastroesophageal junction, *EUS* Echo-endoscopy

A transthoracic/transhiatal esophagectomy with lymphadenectomy was performed after a median time of 91.5 days (range: 41–148 days) after completion of nCRT. A complete resection (R0) was performed in all patients (Table 2).

**Table 2:** Surgical specimen characteristics

Patient	Tumor length (mm)	Histology	Contraction rate (%)	Post-process contraction rate (%)	Resection margin	Mandard	Macroscopic tumor beyond EDTB (mm)		Microscopic tumor beyond EDTB (mm)	
							Cranial	Caudal	Cranial	Caudal
1	52	AC	92.2	nd	R0	4	0	0	0	0
2	55	AC	89.1	95.9	R0	3	0	0	0	0
3	46	AC	95.7	104.5	R0	3	0	0	0	0
4	45	AC	95.6	97.7	R0	2	na	na	0	0
5	38	AC	89.5	101.5	R0	1	na	na	0	0
6	21	AC	101.4	nd	R0	4	0	0	0	0
7	54	AC	96.3	nd	R0	3	na	na	0	15
8	66	AC	98.8	98.6	R0	3	na	na	0	10
9	15	SCC	92.0	nd	R0	1	na	na	0	0
10	45	AC	94.4	nd	R0	4	0	0	0	0
11	65	AC	88.6	97.7	R0	2	na	na	0	15^b^
12	72	SCC	92.3	98.5	R0	3	0	0	8	0
13	80	AC	93.0	97.8	R0	3	na	na	0	0
14	51	AC	92.9	102.3	R0	3	na	na	5	5
15^a^	88	AC	na	nd	R0	3	0	na	0	na
16	36	AC	97.8	nd	R0	2	na	na	0	0
17	51	SCC	98.0	99.6	R0	1	na	na	0	0
18	22	SCC	95.8	nd	R0	1	na	na	0	0
19^a^	37	AC	na	nd	R0	2	0	na	0	na
20	27	AC	97.8	nd	R0	4	0	0	0	0
21	84	AC	94.5	nd	R0	3	na	na	0	0
22	96	SCC	83.3	nd	R0	2	na	na	0	0
23	20	AC	91.0	nd	R0	1	na	na	0	0
24^a^	98	AC	na	nd	R0	4	0	na	6	na
25	43	AC	95.6	nd	R0	3	na	na	0	0
26	79	AC	na	nd	R0	2	na	na	0	0
27^a^	52	AC	92.7	nd	R0	3	na	na	na	0
28	40	SCC	92.1	nd	R0	2	na	na	0	0
29	29	SCC	91,7	nd	R0	1	na	na	0	0
30	12	AC	85,2	nd	R0	1	na	na	0	0
31	30	SCC	93,8	nd	R0	1	na	na	0	0
32	40	SCC	93,0	nd	R0	1	na	na	0	0

Patient without fiducial markers on pre-operative imaging excluded from table

*Abbreviations*: *EDTB* (echo)endoscopically determined tumor border, *AC* Adenocarcinoma, *SCC* Squamous cell carcinoma, *nd* not determined, *na* not applicable, *R0* Complete resection, *mm* millimeters. Presented tumor spread is uncorrected for contraction rate

^a^Only one EDTB available for evaluation. ^b^ Only regressional changes visible, no viable tumor cells

Of the 33 patients, 28 patients had ≥2 fiducial markers in situ visible on the post-operative CT scan, allowing inspection of the cranial and caudal EDTB. In 4 patients, only 1 fiducial marker was visible on the post-operative CT scan, only allowing inspection of one EDTB. One patient lost all fiducial markers and was excluded from further analysis. In total, 60 EDTBs were examined in 32 patients.

### Contraction rate

To adjust for specimen contraction ex-vivo, we calculated the contraction rate per specimen. In four patients, only one fiducial marker was present on CT scan, hampering individual contraction rate calculation. The contraction rate of the esophageal specimens ranged from 83.3 to 101.4%, with a mean value of 93.2% + 4.4% (SD).

In a subset of patients (*n* = 10), the possible *processing* contraction rate after formalin processing was also calculated, but not included in the adjustment for contraction. A mean value of 99.4% + 2.6% (SD) was found, validating the absence of post-processing contraction with modern processing techniques.

### Accuracy of markers for demarcation of macroscopic tumor spread

Macroscopically evident residual tumor was found in 10 patients (31.3%) and was located between both EDTB’s in 7 patients. In the other 3 patients, with only a cranial EDTB available for inspection, no macroscopic spread was found beyond the cranial EDTB.

### Microscopic tumor spread beyond EDTB

A complete microscopic response of the primary tumor (ypT0) was seen in nine patients (28.1%). Fifty percent (16/32) of patients had a Mandard 3 or higher (i.e., residual tumor group), which could be analysed for MS (Table 2).

In the residual tumor group, 5/16 (31.3%) patients showed MS beyond the EDTB. 3/15 (20%) patients had MS beyond the cranial EDTB, and 3/14 (21%) had MS beyond the caudal EDTB. The cranial MS ex-vivo was 8, 5, and 6 mm in longitudinal direction. Adjusted for contraction this resulted in 8.6 mm, 5.4 mm, and 6.5 mm in situ, respectively. For the last patient with an MS of 6 mm, an individual contraction rate could not be determined, since only 1 fiducial marker was visible on post-operative CT imaging, instead the mean contraction rate was used for in situ MS calculation.

The caudal MS ex-vivo was 15, 10, and 5 mm in longitudinal direction, resulting in-situ in 15.6 mm, 11.7 mm, and 5.4 mm, respectively.

Of the complete/partial responding group (i.e., Mandard 1–2); one patient with a Mandard 2 showed some regressional changes (i.e., mucinous lakes) crossed the caudal EDTB by 15 mm (17.9 mm, adjusted for contraction). Pathological findings are listed in Table 2. In univariate analysis only tumor length was associated with MS beyond EDTB (*p* = 0.028). This association did not remain significant after multivariate analysis.

### DFS and OS

The median follow-up time was 29.5 months (IQR: 17.8–38.6 months). Fifty percent (16/32) of patients had locoregional and/or distant recurrences. The most frequently reported first site of recurrence was distant (94%). Only one patient developed a strict locoregional recurrence at a regional lymph node station outside the radiation field (6%). The 2- and 1-year OS was 70.3 and 84.4%, respectively. The 2- and 1-year DFS was 60.4 and 87.1%.

## Discussion

Up to date, this is the first study to quantify MS beyond EDTBs in esophageal cancer patients that underwent nCRT. Our results demonstrate that fiducial markers placed on EDTB are a good surrogate for macroscopic gross tumor extent, with no macroscopic tumor spread seen in the surgical specimen beyond the EDTB. Furthermore, when fiducial markers placed on the EDTB are used to determine the GTV, we found a maximum MS of 9 mm cranially and 16 mm caudally beyond the GTV.

MS beyond macroscopic tumor borders of esophageal tumors has been previously investigated in four studies. Three of these studies assessed only SCC, whereas adenocarcinoma has been investigated in only one study [7–9, 14]. The general procedure of these studies was to determine the macroscopic tumor on the ex-vivo resection specimen, and subsequently examine it for MS along the length of the esophagus, taking tissue contraction into account by numerous strategies, ranging from stretching the specimen to its in-situ length and pinning it to a board to simply correcting measurements by a contraction factor determined for the specimen as a whole. These studies recommended CTV margins extending 3 cm cranially and 4–5 cm caudally from the GTV to cover 95% of all MS of the primary tumor [7, 9].

With a maximum extent of 9 mm cranially and 16 mm caudally, the MS found in our study is less than previously described. There are several explanations for this. First, there might be a discrepancy in used contraction rate. Gao et al. found a contraction rate ranging from 22.0 to 96.0%, (mean = 54.6%). In their study a 5/3-cm long piece of healthy esophagus was used to determine the contraction rate, defined as the length measured on the hematoxylin- and eosin-stained slide divided by the length measured in situ before the surgery with a ruler. The same method was used in a more recent study by Song et al., nonetheless this method is controversial since a different shortening has been reported earlier of the healthy esophageal ends (i.e., contraction rate + 30%) in comparison to the central tumorous esophagus, which retains 90% of its original length [15]. Subsequently, an overestimation of the contraction rate by Gao et al. when using the healthy esophagus as a reference is imaginable, resulting in a substantial overestimation of MS. In our study, the presence of the fiducials made it possible to calculate a contraction rate representative for the true tumorous tissue. We found a mean contraction rate of 93%, similar to the one of reported by Siu et al. [15]. Further post-processing contraction was successfully prevented by pinning the esophagus to a paraffin board, as was demonstrated in a subset of our patients.

Secondly, in these earlier studies MS was assessed in relation to the ex-vivo determined macroscopic tumor, which might not be representative for currently in vivo determined GTV with the aid of (echo)endoscopic information and fiducial markers placed on EDTB. Endoscopic examination is still considered the most reliable tool for assessment of macroscopic tumor extension, and correlates significantly with pathological tumor extension [16, 17]. Conventional endoscopy can assess the location, mucosal appearance, and consistency of an esophageal tumor but cannot provide information on submucosal extent. Therefore, endoscopic ultrasonography (EUS) has emerged as the most reliable method for evaluating submucosal tumors [18]. EUS accuracy only has been investigated in terms of diagnostic performance instead of spatial performance, however our findings (i.e., much smaller MS than in previous studies) might also suggest that EUS might include parts of the microscopic disease (i.e., the CTV) as well.

Lastly, and also the largest and foremost limitation of our study is the use of nCRT. The effect of the nCRT found in our study, was comparable to the regression grade found in earlier nCRT studies [12]. Since the tumor regression grade of the primary tumor is significantly related to the risk of residual tumor cells in the esophageal wall, only tumors with no response or only a partial response (Mandard 3 or higher) were analyzed for MS beyond EDTB [12]. Nonetheless, the use of nCRT might underestimate the MS in case nCRT did completely eradicate MS but not the primary tumor. Furthermore, it cannot be excluded that Mandard 3–5 tumors might be a selection of tumors with a less extensive microscopic growth pattern, although the opposite is more likely. Tsutsui et al. investigated MS in 303 patients with pT1–4 squamous cell carcinoma treated with nCRT in 92% of cases. In this study, they observed MS proximal and distal to the tumor in + 60% of cases and still found MS with a maximal extent of 30–106 mm, despite the nCRT, which is in line with the other pathology studies using a similar methodology. In a more recent article by Muijs et al., 14% of patients treated with nCRT showed residual MS even outside the CTV (i.e., 35 mm beyond the GTV) [19]. In the latter study, the GTV and CTV were reconstructed using anatomical reference points. This less accurate methodology of GTV determination could have led to the marked amounts of MS (MS even larger than found in the Asian studies). The lack of fiducial markers could have led to severe geometric misses as was shown in a study comparing GTV delineation with and without fiducial markers [4]. Both studies demonstrate that similar ranges of MS extent are found as in untreated specimens, lessening the probability of a nCRT-effect in our findings.

Another peculiar finding is the more pronounced MS caudally, compared to the cranial MS. In multivariate analysis no predictors were found, albeit since numbers are very small this analysis might not be insightful. The two patients with an MS of 16 and 10 mm caudally, had an intestinal type junction tumor and diffuse-type distal esophageal tumor, respectively.

Our findings suggest that current CTV margins might be too large to account for the MS in the esophageal wall. In selected patients treated with nCRT, in absence of pathological lymph nodes and in which no elective node irradiation is indicated or considered too toxic, CTV margins might be reduced to 1 cm in cranial and 1.5 cm in caudal direction. The European Organization for Research and Treatment of Cancer guidelines for nCRT of adenocarcinoma at the GEJ recommended earlier a minimal CTV margin of 1.5 cm caudally and a 1-cm margin cranially, in line with our findings when fiducial markers are used [20]. They did not, however, provide a scientific substantiation on how they came to this conclusion. Since the CTV is not only determined by tumor spread in the esophageal wall but it is also considered necessary to include an elective or pathologic node area, in combination with the use of nCRT in our study and the small sample size, our findings have no direct effect on the clinically applied treatment volume in general.

In a definitive setting, reduction of CTV margins might be too preliminary. Especially since the literature appears to indicate that the main pattern of RT failure is in-field recurrence, while the field-edge and out-field recurrences are rare [21, 22]. Our findings could be important for determination of a GTV-to-CTV margin in case a boost is given in a definitive CRT setting. In that case a boost GTV-to-CTV margin of more than 1 cm cranially is unnecessary to compensate for MS. In order to reduce toxicity of the boost dose, a caudal margin of 1 cm might also be appropriate, since in the vast majority of our patients the MS extension was restricted to 10 mm beyond the macroscopic border. Results of a recently completed boost trial in the Netherlands have to be awaited (http://www.trialregister.nl/trialreg/admin/rctview.asp?TC=3532).

In summary, no macroscopic tumor was found beyond EDTBs. Only 20 and 21% of the cranial and caudal EDTB respectively, were crossed with a maximum of 9 mm and 16 mm MS in the residual tumor group. Our results validate the use of fiducial markers on EDTB as a surrogate for macroscopic tumor and indicate that current recommended CTV margins around the GTV to compensate for microscopic tumor along the esophageal wall can be reduced when the GTV is determined with the aid of fiducial markers. As yet, currently applied GTV-to-CTV margins cannot be limited on the basis of this study, since subclinical disease in regional lymph nodes remains an additional factor determining the CTV volume.

## Data Availability

The datasets used and/or analysed during the current study are available from the corresponding author on reasonable request.

## Abbreviations

AD: Adenocarcinoma
CBCT: Cone beam CT
CC: Craniocaudal
CTV: Clinical target volume
DFS: Disease-free survival
EDTB: (echo)endoscopically determined tumor borders
EUS: Echo-endoscopy
FDG-PET/CT: 18-F-Fluorodeoxyglucose positron emission tomography/computer tomography
GTV: Gross tumor volume
IMRT: Intensity-modulated RT
MS: Microscopic tumor spread
nCRT: neo-adjuvant chemoradiotherapy
OS: Overall survival
PTV: Planning target volume
RT: Radiotherapy
SCC: Squamous cell carcinoma
VMAT: Volumetric modulated arc therapy
